# Wnt and Vitamin D at the Crossroads in Solid Cancer

**DOI:** 10.3390/cancers12113434

**Published:** 2020-11-19

**Authors:** José Manuel González-Sancho, María Jesús Larriba, Alberto Muñoz

**Affiliations:** 1 Instituto de Investigaciones Biomédicas “Alberto Sols”, Consejo Superior de Investigaciones Científicas, Universidad Autónoma de Madrid, 28029 Madrid, Spain; josemanuel.gonzalez@uam.es (J.M.G.-S.); mjlarriba@iib.uam.es (M.J.L.); 2 Departamento de Bioquímica, Facultad de Medicina, Universidad Autónoma de Madrid, 28029 Madrid, Spain; 3 Centro de Investigación Biomédica en Red de Cáncer (CIBERONC), 28029 Madrid, Spain; 4 Instituto de Investigación Sanitaria del Hospital Universitario La Paz—IdiPAZ (Hospital Universitario La Paz—Universidad Autónoma de Madrid), 28029 Madrid, Spain

**Keywords:** wnt, β-catenin, vitamin D, cancer, colon cancer

## Abstract

**Simple Summary:**

The Wnt/β-catenin signaling pathway is aberrantly activated in most colorectal cancers and less frequently in a variety of other solid neoplasias. Many epidemiological and experimental studies and some clinical trials suggest an anticancer action of vitamin D, mainly against colorectal cancer. The aim of this review was to analyze the literature supporting the interference of Wnt/β-catenin signaling by the active vitamin D metabolite 1α,25-dihydroxyvitamin D_3_. We discuss the molecular mechanisms of this antagonism in colorectal cancer and other cancer types. Additionally, we summarize the available data indicating a reciprocal inhibition of vitamin D action by the activated Wnt/β-catenin pathway. Thus, a complex mutual antagonism between Wnt/β-catenin signaling and the vitamin D system seems to be at the root of many solid cancers.

**Abstract:**

Abnormal activation of the Wnt/β-catenin pathway is common in many types of solid cancers. Likewise, a large proportion of cancer patients have vitamin D deficiency. In line with these observations, Wnt/β-catenin signaling and 1α,25-dihydroxyvitamin D_3_ (1,25(OH)_2_D_3_), the active vitamin D metabolite, usually have opposite effects on cancer cell proliferation and phenotype. In recent years, an increasing number of studies performed in a variety of cancer types have revealed a complex crosstalk between Wnt/β-catenin signaling and 1,25(OH)_2_D_3_. Here we review the mechanisms by which 1,25(OH)_2_D_3_ inhibits Wnt/β-catenin signaling and, conversely, how the activated Wnt/β-catenin pathway may abrogate vitamin D action. The available data suggest that interaction between Wnt/β-catenin signaling and the vitamin D system is at the crossroads in solid cancers and may have therapeutic applications.

## 1. Introduction

### 1.1. Wnt

Wnt proteins are extracellular signaling molecules that control many key processes during embryonic development and regulate the homeostasis of adult tissues, mainly by modulating the survival, self-renewal, and proliferation of stem cells. They are secreted by a variety of cell types and typically have a short range of action, mediating communication between neighboring cells. Wnt proteins bind to cell surface receptors, of which several classes have been described. Specific Wnt-receptor combinations and cellular contexts determine which of the existing Wnt signaling pathways is engaged [1].

The Wnt/β-catenin pathway is triggered by Wnt binding to cell membrane receptors of the Frizzled and low-density lipoprotein receptor-related protein (LRP) families. In the absence of a Wnt ligand, β-catenin protein is mainly located at cell-cell contacts and free cytoplasmic β-catenin is kept low because of a proteolytic destruction machinery. A complex containing the tumor suppressor proteins APC (*adenomatous polyposis coli*) and axin, and the kinases casein kinase 1 (CK1) and glycogen synthase kinase-3β (GSK-3β) targets β-catenin for *N*-terminal phosphorylation and subsequent ubiquitination and proteasome-mediated degradation. Wnt binding to Frizzled and LRP5/6 leads to inhibition of the β-catenin destruction complex and, therefore, to the accumulation of β-catenin in the cytoplasm. A proportion of β-catenin enters the nucleus and binds to transcription factors of the LEF/TCF family acting as a co-activator and regulating the expression of a large variety of genes. Wnt target genes are cell and tissue type-dependent and affect many cellular functions and processes, such as cell proliferation, stemness, migration, and invasion. Some of these targets include the *c*-*MYC* and *CCND1*/cyclin D1 oncogenes, the Wnt inhibitors *DKK1* and *NKD1/2*, and the Wnt effector *LEF1*. In addition, the Wnt inhibitor *AXIN2* is the most ubiquitously regulated β-catenin target gene [1,2,3].

Wnt/β-catenin signaling is highly dependent on the number of Frizzled receptor molecules present on the cell surface. Vertebrates have evolved a complex regulatory mechanism to control the amount of Frizzled on the plasma membrane that involves three types of proteins: leucine-rich repeat-containing G-protein coupled receptors (LGR4-6), their extracellular ligands R-spondins (RSPO1-4), and the E3 transmembrane ubiquitin ligases ZNRF3 and RNF43 [4,5]. In the absence of RSPO, Frizzled receptors are targeted for degradation by ZNRF3/RNF43-mediated ubiquitination, which results in low Frizzled membrane concentration and, therefore, in attenuated Wnt signaling. In contrast, RSPO binding to LGR4-6 sequesters ZNRF3/RNF43 in a ternary complex and prevents ubiquitin tagging of Frizzled. Thus, RSPOs are responsible for the accumulation of Frizzled receptors on the cell surface and the potentiation of Wnt/β-catenin signaling in target cells [6,7,8].

Dysregulation of Wnt/β-catenin signaling is involved in human diseases including cancer. In many types of cancer, e.g., colorectal, breast, and liver carcinoma, melanoma and leukemia, β-catenin constitutively accumulates within the nucleus of tumor cells [9,10,11,12,13]. In fact, aberrant activation of the Wnt/β-catenin pathway is the most common event in human colorectal cancer (CRC) [14,15] in which massive sequencing has estimated that over 94% of primary colon tumors carry mutations in one or more genes involved in this pathway [16]. Truncation mutations and allelic losses in the tumor suppressor gene *APC* are present in around 80% of sporadic CRC cases, whereas a small proportion carries mutations in *AXIN2* or *CTNNB1*/β-catenin genes. Moreover, chromosomal rearrangements in R-spondin family members *RSPO2* and *RSPO3* have been found in 10% of human CRC leading to enhanced Wnt signaling [17,18]. In addition, *RNF43* is mutated in a proportion of mismatch repair-deficient colon tumors. Alterations in genes encoding components of the Wnt/β-catenin pathway are frequently mutually exclusive, which confirms that aberrant activation of this pathway is a hallmark of CRC. Mutations in *CTNNB1*/β-catenin or *AXIN2* have been reported in other human tumors, e.g., hepatocellular carcinomas [19,20,21], whereas overexpression of Wnt factors/receptors or silencing of extracellular Wnt inhibitors are the preferred mechanisms of Wnt/β-catenin sustained activation in other cancers, e.g., breast and lung cancers [22,23,24,25,26,27].

### 1.2. Vitamin D

Vitamin D_3_ (cholecalciferol) is a natural seco-steroid whose main source is non-enzymatic production in human skin from UV-B exposed 7-dehydrocholesterol, an abundant cholesterol precursor [28,29]. Vitamin D_3_ from skin production and from dietary uptake is hydroxylated in the liver to 25-hydroxyvitamin D_3_ (25(OH)D_3_, calcidiol), a stable compound that is used as a biomarker for the vitamin D status of a person [29,30,31]. Subsequent hydroxylation of 25(OH)D_3_ at carbon 1, which occurs mainly in the kidneys but also in several types of epithelial and immune cells, renders 1,25-dihydroxyvitamin D_3_ (1,25(OH)_2_D_3_, calcitriol). This is the most active vitamin D_3_ metabolite and a high affinity ligand for the vitamin D receptor (VDR) [29,31,32].

VDR is a member of the nuclear receptor superfamily of transcription factors, which includes receptors for other hormones such as estrogen, progesterone or glucocorticoids, as well as a number of orphan receptors. Nuclear receptors present a highly conserved ligand-binding domain, which in the case of VDR fixes 1,25(OH)_2_D_3_ or its synthetic analogues with high specificity [33]. Binding of 1,25(OH)_2_D_3_ to VDR promotes the formation of complexes with RXR, the receptor for 9-*cis*-retinoic acid, and the binding of these VDR/RXR heterodimers to DNA. This leads to epigenetic changes that affect the transcription rate of hundreds of target genes involved in many cellular processes, including proliferation, differentiation and survival [29,31]. Moreover, a proportion of VDR molecules locate in the cytoplasm of some cell types where, on ligand binding, they trigger rapid, non-genomic, modulatory effects on signaling pathways by acting on kinases, phosphatases, and ion channels [34,35].

Current evidence indicates that 1,25(OH)_2_D_3_ and its derivatives modulate signaling pathways that affect cell survival, growth, and differentiation [29,36,37], key processes that are dysregulated in human cancers. One of these signaling routes is the Wnt/β-catenin pathway. This review will focus on the crosstalk between Wnt and vitamin D in solid tumors.

## 2. Antagonism of Wnt/β-Catenin Signaling by 1,25(OH)_2_D_3_ in Solid Cancers

### 2.1. Colorectal Cancer

Four decades ago, an epidemiological study hinted at the protective effects of vitamin D_3_ against CRC by indicating that high UVB exposure or life at lower latitudes, both of which result in higher vitamin D_3_ synthesis, lead to lower incidence of CRC [38]. Since then, a large number of epidemiological studies, experimental work performed in cultured cells and animal models, and also some, but not all, vitamin D_3_ supplementation human clinical trials have strongly suggested that 1,25(OH)_2_D_3_ has anticancer effects, particularly in CRC [31,37,39,40,41,42,43,44].

Our group was a pioneer in demonstrating that 1,25(OH)_2_D_3_ antagonizes the Wnt/β-catenin signaling pathway in colon carcinoma cells [45], a mechanism that could at least partly account for the protective effects of vitamin D_3_ observed in epidemiological and animal studies. Previously, other groups had reported a crosstalk between Wnt signaling and other nuclear receptors, such as those for retinoid acid and androgen [46,47].

**Figure 1:**
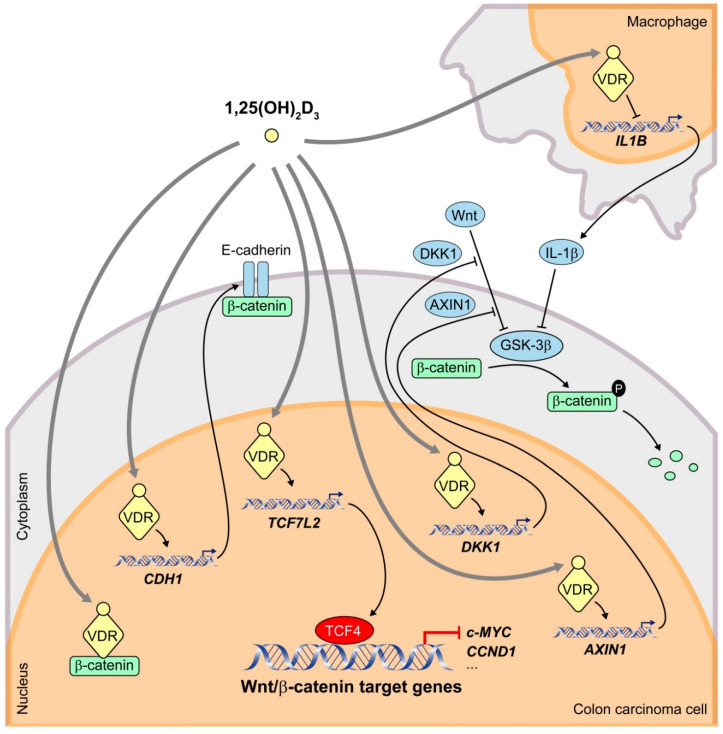
Schematic representation of the mechanisms by which 1,25(OH)_2_D_3_ interferes the Wnt/β-catenin signaling pathway in human CRC cells. 1,25(OH)_2_D_3_ binds to its high affinity receptor VDR inducing the formation of β-catenin/VDR complexes and thus preventing that of transcriptionally active β-catenin/TCF4 complexes. In addition, 1,25(OH)_2_D_3_ increases the transcription of the *CDH1* gene encoding E-cadherin, which sequesters newly synthesized β-catenin protein at the subcortical adherens junctions. Furthermore, 1,25(OH)_2_D_3_ upregulates the expression of the negative regulators of the Wnt/β-catenin pathway *TCF7L2* (encoding TCF4), *DKK1* and *AXIN1*. 1,25(OH)_2_D_3_ also antagonizes the pathway by reducing the secretion by nearby macrophages of IL-1β, which inhibits GSK-3β activity in CRC cells leading to an increase in β-catenin levels.

Results from our laboratory showed that 1,25(OH)_2_D_3_ interferes with Wnt/β-catenin signaling in human colon carcinoma cells by at least three mechanisms (Figure 1). Firstly, ligand-activated nuclear VDR binds and sequesters β-catenin, which prevents its binding to LEF/TCF transcription factors and thus blocks β-catenin/TCF-mediated transcription of Wnt target genes [45]. VDR/β-catenin physical interaction was later confirmed in this and other cell systems and involves the *C*-terminal region of β-catenin and the activator function-2 domain of VDR [48]. Interestingly, wild-type APC potentiates VDR/β-catenin binding [49]. Lithocholic acid, a low affinity VDR ligand, also prompts this interaction, although less efficiently than 1,25(OH)_2_D_3_ [49].

Secondly, 1,25(OH)_2_D_3_ induces the expression of E-cadherin protein, which sequesters newly synthesized β-catenin at subcortical cell-cell adherens junctions, thus avoiding its translocation to the nucleus and β-catenin/TCF-mediated transcription [45]. Our data suggest that the small GTPase RhoA, the protease inhibitor cystatin D, the regulator of tyrosine kinase receptor signaling Sprouty-2, and the histone demethylase JMJD3 are involved in this mechanism [34,50,51,52]. Induction of E-cadherin by 1,25(OH)_2_D_3_ and concomitant inhibition of the Wnt/β-catenin pathway have also been reported in other cell types [53]. However, 1,25(OH)_2_D_3_ can antagonize Wnt/β-catenin signaling in colon carcinoma cells that do not express E-cadherin, which implies that this mechanism is not strictly required [45].

Thirdly, 1,25(OH)_2_D_3_ promotes the expression of Dickkopf 1 (DKK1), a member of a family of extracellular inhibitors of the Wnt/β-catenin pathway [54]. DKK1 can inhibit Wnt/β-catenin signaling by two mechanisms. On the one hand, DKK1 direct binding to LRP5/6 blocks Wnt-LRP interaction [55]. On the other, DKK1 can engage a ternary complex with LRP5/6 and Kremen receptors, which prompts rapid endocytosis and removal of LRP5/6 from the plasma membrane [56]. In addition to a Wnt inhibitor, *DKK1* is a β-catenin/TCF target gene [57,58,59]. Although most CRC carry mutations that activate the Wnt/β-catenin pathway downstream of DKK1, evidence suggests that this extracellular inhibitor has antitumor effects that are independent of β-catenin/TCF transcriptional activity [60,61,62]. Supporting the relevance of DKK1 in CRC, we and others have demonstrated that DKK1 expression is frequently downregulated in this neoplasia [59], in part due to gene promoter hypermethylation [62,63,64]. Moreover, the expression levels of VDR and DKK1 in human CRC biopsies directly correlate [54], and dietary vitamin D intake is inversely associated with *DKK1* promoter methylation in a large cohort of CRC patients [65].

DKK4 is another member of the Dickkopf family of Wnt extracellular inhibitors, although it is a weaker Wnt antagonist than DKK1. We reported that 1,25(OH)_2_D_3_ downregulates the expression of DKK4 in both human colon and breast cancer cells. Accordingly, a significant inverse correlation between DKK4 and VDR expression exists in human CRC biopsies [66]. Unexpectedly, overexpression of DKK4 in human CRC cells enhances their migratory, invasive, and angiogenic potential [66]. These effects are probably unrelated to Wnt/β-catenin inhibition and imply additional mechanisms of action of DKK4. In this regard, we and others found that DKK4 transcripts are overexpressed in human CRC samples and in biopsies from patients with inflammatory bowel disease [66,67,68]. These data suggest that downregulation of DKK4 by 1,25(OH)_2_D_3_ may be another mechanism for the antitumor action of 1,25(OH)_2_D_3_ in CRC.

The *c*-*MYC* oncogene is a well-known β-catenin/TCF target gene that is frequently deregulated in human cancers and activates genetic programs that orchestrate biological processes to promote cell growth and proliferation [69]. Therefore, targeting the function of MYC oncoproteins holds the promise of achieving new, effective anticancer therapies that can be applied to a broad range of tumors [70]. In particular, mutational and integrative analyses have stressed the essential role of *c*-*MYC* in CRC [16]. A study reported by Meyer and colleagues using chromatin immunoprecipitation assays followed by high-throughput DNA sequencing (ChIP-Seq) in the CRC cell line LS180 concluded that β-catenin/TCF4 and VDR/RXR heterodimers colocalize at 74 sites near a limited set of genes that included *c*-*MYC* and *c*-*FOS* oncogenes [71]. These data strongly suggest a direct action of both complexes at these gene *loci*. In fact, 1,25(OH)_2_D_3_ effects on *c*-*MYC* gene expression may count as another mechanism of crosstalk between 1,25(OH)_2_D_3_ and Wnt/β-catenin signaling pathways. Firstly, ligand-activated VDR represses *c*-*MYC* expression by direct interaction with two vitamin D response elements (VDRE) in the promoter region [71,72]. Secondly, the antagonism exerted by 1,25(OH)_2_D_3_ on Wnt/β-catenin signaling impairs the transcription of *c*-*MYC* mediated by β-catenin/TCF complexes through their binding to several Wnt responsive elements (WRE) at the *c*-*MYC* promoter [45,73].

Some authors have proposed additional mechanisms of 1,25(OH)_2_D_3_ crosstalk with Wnt/β-catenin signaling in CRC cells (Figure 1). Beildeck and colleagues showed that 1,25(OH)_2_D_3_ increases TCF4 expression in several human CRC cell lines. The effect is indirect but completely dependent on VDR [74]. Tang and colleagues have reported that TCF4 functions as a transcriptional repressor that restricts CRC cell growth [75]. Therefore, 1,25(OH)_2_D_3_/VDR-mediated upregulation of TCF4 possibly has a protective effect on CRC. Furthermore, 1,25(OH)_2_D_3_/VDR induces expression of the negative regulator of the Wnt/β-catenin pathway AXIN1 in CRC cells through a VDRE localized in the regulatory region of the gene [76]. In addition, Gröschel and colleagues found that 1,25(OH)_2_D_3_ reduces nuclear β-catenin levels in LT97 colon microadenoma cells and thus downregulates the expression of Wnt target genes such as *BCL2*, *CCND1*/cyclin D1, *SNAI1*, *CD44*, and *LGR5* [77]. Moreover, in healthy colon of mice fed a high vitamin D diet, β-catenin protein expression is decreased and the same effect is observed for TCF4 [77], which contrasts with the results of Beildeck and colleagues [74].

Kaler and colleagues described a paracrine mechanism that involves not only a crosstalk between 1,25(OH)_2_D_3_ and Wnt/β-catenin signaling pathways but also between carcinoma cells and the tumor microenvironment (Figure 1). They demonstrated that colon carcinoma cells induce the release of interleukin-1β (IL-1β) from macrophagic THP-1 cells in a process that requires constitutive activation of STAT1 [78]. Secreted IL-1β then acts on colon carcinoma cells where it triggers the inactivation of GSK-3β and thus the stabilization of β-catenin and subsequent expression of Wnt target genes. 1,25(OH)_2_D_3_ interrupts this crosstalk by blocking the constitutive activation of STAT1 and thus the production of IL-1β in macrophages in a VDR-dependent manner, which hampers the ability of macrophages to activate Wnt/β-catenin signaling in CRC cells [78]. The possibility that this mechanism works in vivo with tumor-associated macrophages is highly interesting.

Our group has also studied the interplay between 1,25(OH)_2_D_3_ and Wnt3A (an activator of the Wnt/β-catenin pathway) in human colon fibroblasts. Both agents strongly modulate the gene expression profile and phenotype of these cells. However, in contrast to the antagonism exerted by 1,25(OH)_2_D_3_ on the Wnt/β-catenin pathway in colon carcinoma cells, they have a partially overlapping effect. Both compounds inhibit fibroblast proliferation and migration, but while 1,25(OH)_2_D_3_ reduces, Wnt3A increases fibroblast capacity to remodel the extracellular matrix [79]. In addition, in contrast to the effects observed in established colon carcinoma cell lines, 1,25(OH)_2_D_3_ does not affect the expression of key genes of the Wnt/β-catenin pathway (*AXIN2*, *CCND1*, *DKK1* and *c*-*MYC*) in human colon tumor or normal organoids derived from CRC patients, where only the *DKK4* Wnt/β-catenin target gene is repressed by 1,25(OH)_2_D_3_ [80]. This shows that antagonism of the Wnt/β-catenin pathway is not a universal action of 1,25(OH)_2_D_3_ in tumor contexts. Moreover, 1,25(OH)_2_D_3_ cooperates with Wnt factors in the differentiation of bone (osteoblasts), skin (keratinocytes) and brain (neuronal precursors) cells under physiologic conditions [81,82]. Together, available data indicate a mostly repressive action of 1,25(OH)_2_D_3_ on overactivation of the Wnt/β-catenin pathway with different effects in particular scenarios.

The interplay between 1,25(OH)_2_D_3_ and Wnt/β-catenin signaling in CRC has also been studied in vivo in animal models and patients. Our group showed that the 1,25(OH)_2_D_3_ analogue EB1089 reduces the growth of xenografts generated by human CRC cells in immunosuppressed mice. In line with data obtained in cell cultures, this inhibition is associated with an increase of E-cadherin and DKK1 levels, and a decrease of β-catenin nuclear content and of the expression of the β-catenin/TCF target gene *ENC1* in the xenografts [54,83,84]. Likewise, the antitumor action of 1,25(OH)_2_D_3_ on chemically induced mouse intestinal tumors is concomitant with increased expression of E-cadherin and the inhibition of β-catenin/TCF target genes such as *c*-*Myc* and *Ccnd1*/cyclin D1 in the intestinal crypts of these animals [85,86]. Concordantly, Xu and colleagues reported that 1,25(OH)_2_D_3_ and two of its analogues reduce the tumor load in the *Apc*^min/+^ mouse model of intestinal tumorigenesis associated with an increase of E-cadherin protein and a decrease of nuclear β-catenin levels and of the expression of the Wnt target genes *c*-*Myc* and *Tcf1* [87].

A Western-style diet that is high in fat and low in calcium and vitamin D is a risk factor for gastrointestinal carcinogenesis. This diet increases the frequency of intestinal tumors in normal mice and speeds up tumor formation in mouse models for intestinal cancer [88]. Several groups have shown that a Western-style diet alters components of the Wnt/β-catenin pathway in intestinal epithelial cells of normal mice [88,89]. These effects can be reversed by calcium and vitamin D supplementation, which prevents the increase of β-catenin/TCF transcriptional activity and reduces the expression of β-catenin, Ephb2 and Frizzled-2, -5, and -10 [89,90].

*Vdr* knockout mice have also been used to study the role of the vitamin D pathway on CRC. Larriba and colleagues [91] and Zheng and colleagues [92] generated *Apc*^min/+^ *Vdr*^−/−^ mice and discovered that the absence of Vdr results in a higher tumor load and an increased number of premalignant lesions. Interestingly, nuclear staining of β-catenin and expression of Wnt target genes *Ccnd1*/cyclin D1 and *Lef1* are higher in *Apc*^min/+^ *Vdr*^−/−^ than in *Apc*^min/+^ *Vdr*^+/+^ mice. This suggests that *Vdr* inactivation facilitates intestinal tumorigenesis fostered by Wnt/β-catenin activation [91,92].

Remarkably, in a randomized, double-blinded, placebo-controlled clinical trial, Bostick’s group reported that vitamin D supplements increase the expression of APC, E-cadherin and other differentiation markers, and decrease that of β-catenin in the upper part of the crypt of normal rectal mucosa from sporadic colorectal adenoma patients [93,94,95,96]. In addition, a recent study with 67 CRC patients has revealed that a high circulating 25(OH)D_3_ level associates with low promoter methylation of secreted frizzled-related protein 2 (*SFRP2*) gene that encodes a soluble inhibitor of the Wnt/β-catenin pathway [97]. These data support an inhibitory effect of vitamin D on Wnt signaling in the human colon in vivo.

### 2.2. Other Solid Tumors

Although Wnt signaling was first described as inducing breast tumors in mice [98] and Wnt/β-catenin signaling is activated in a proportion of multiple subtypes of human breast cancers [10,99], the typical mutations in components of the pathway found in CRC (*APC*, *CTNNB1*, *AXIN2*) are rare in breast carcinomas [27]. The elevated level of nuclear β-catenin and Wnt signaling in these tumors may be due to high expression of Wnt factors in the tumor environment, loss of APC, Wnt inhibitors (DKK1, SFRPs), and/or E-cadherin expression by epigenetic modification/gene silencing, or alterations in the expression of other genes that encode constituents of the pathway (*RSPO2*, *FZD6*) [27].

Notably, nuclear β-catenin accumulation in a subset of triple-negative and basal-like breast cancer subtypes has been associated with a poor patient outcome [10,99]. Our group has shown that 1,25(OH)_2_D_3_ downregulates the expression of myoepithelial/basal markers, such as P-cadherin, smooth muscle α-actin, and α6 and β4 integrins in a panel of breast carcinoma cells, and that *Vdr*^−/−^ mice express higher levels of P-cadherin and smooth muscle α-actin in the mammary gland than *wt* littermates [100]. These results suggest that 1,25(OH)_2_D_3_/VDR antagonizes the Wnt/β-catenin pathway in breast cancer cells, which might protect against the triple-negative and basal-like phenotype. In line with this, 1,25(OH)_2_D_3_ induces DKK1 expression and reduces β-catenin transcriptional activity in R7 murine breast cancer cells, and *Vdr* deletion and 1,25(OH)_2_D_3_ treatment increases and inhibits, respectively, the tumor expression of several Wnt/β-catenin target genes in breast cancer mouse models [101,102]. The capacity of 1,25(OH)_2_D_3_ to inhibit spheroid formation by breast cancer stem cells is overcome by β-catenin overexpression, which suggests that inhibition of the Wnt/β-catenin pathway is essential for this action of 1,25(OH)_2_D_3_ [101]. Furthermore, Zheng and colleagues have reported that VDR overexpression in a stem cell-enriched subpopulation of MCF-7 breast cancer cells inhibits Wnt/β-catenin signaling and increases cell sensitivity to tamoxifen [103]. Surprisingly, however, in another study the stable knockdown of *VDR* expression leads to attenuation of the Wnt/β-catenin pathway in MDA-MB-231 breast cancer cells: cytoplasmic and nuclear levels of β-catenin are reduced with the subsequent downregulation of its target genes *AXIN2*, *CCND1*/cyclin D1, *IL6*, and *IL8* [104].

Long non-coding RNA colon cancer-associated transcript 2 (*CCAT2*) is upregulated in ovarian cancer cells and promotes epithelial-mesenchymal transition (EMT) at least partially through the Wnt/β-catenin pathway. *CCAT2* knockdown represses the expression of β-catenin and the activity of TCF/LEF factors and inhibits EMT by upregulating E-cadherin and downregulating N-cadherin, Snail1, and Twist1 [105]. Of note, 1,25(OH)_2_D_3_ inhibits CCAT2 expression in ovarian cancer cells concomitantly with a reduction in cell proliferation, migration, and invasion. This is linked to decreased binding of TCF4 to the *c*-*MYC* promoter and, thus, to repression of *c*-MYC protein expression [106]. Thus, inhibition of CCAT2 represents a novel mechanism of Wnt/β-catenin antagonism by 1,25(OH)_2_D_3_. In addition, Srivastava and colleagues have shown that 1,25(OH)_2_D_3_/VDR can deplete ovarian cancer stem cells via inhibition of the Wnt/β-catenin pathway [107].

A recent study has investigated whether 1,25(OH)_2_D_3_ can affect Wnt/β-catenin signaling in human uterine leiomyoma primary cells using a Wnt pathway PCR array. Up to 75% of the β-catenin/TCF target genes analyzed are repressed by 1,25(OH)_2_D_3_. Similarly, 1,25(OH)_2_D_3_ inhibits the expression of 73.3% and 77.2% of the Wnt-related genes involved in tissue polarity and cell migration, and in cell cycle, cell growth and proliferation, respectively [108]. These results suggest that not only Wnt/β-catenin but probably also Wnt non-canonical pathways are inhibited by 1,25(OH)_2_D_3_ in this cellular context.

1,25(OH)_2_D_3_ and its analogue TX527 increase β-catenin protein levels in the nucleus and at the plasma membrane in a Kaposi’s sarcoma cellular model and potentiate β-catenin/VDR interaction. The net outcome is downregulation of the β-catenin/TCF target genes *c*-*MYC*, *MMP9* and *CCND1*/cyclin D1. Moreover, VE-cadherin protein and *DKK1* RNA levels are increased [109]. As in Kaposi’s sarcoma cells, 1,25(OH)_2_D_3_ augments the level of total β-catenin (both cytoplasmic and nuclear pools), while it reduces that of phosphorylated β-catenin in renal cell carcinoma cells [110]. More importantly, 1,25(OH)_2_D_3_ enhances VDR/β-catenin interaction while attenuating β-catenin/TCF binding. Accordingly, 1,25(OH)_2_D_3_ downregulates the expression of *CCND1*/cyclin D1 and *AXIN2* genes. In addition, 1,25(OH)_2_D_3_ upregulates E-cadherin expression and blocks TGFβ1-induced nuclear translocation of ZEB1, Snail1 and Twist1, which contributes to the suppression of EMT and the inhibition of cell migration and invasion [110]. Thus, the effects of 1,25(OH)_2_D_3_ on Kaposi’s sarcoma and renal cell carcinoma cells are largely in agreement with those observed on CRC cells. Distinctly, in pancreatic carcinoma cells, the 1,25(OH)_2_D_3_ analogue calcipotriol inhibits Wnt/β-catenin signaling by a different mechanism: the promotion of lysosomal degradation of the Wnt membrane receptor LRP6 [111].

Concomitant with an increase in Wnt/β-catenin signaling, global and epidermal-specific *Vdr* deletion predispose mice to either chemical [112] or long-term UVB-induced [113,114] skin tumor formation. 1,25(OH)_2_D_3_ enhances β-catenin binding to E-cadherin at the plasma membrane, which promotes epidermal cell differentiation. Moreover, VDR competes with LEF/TCF to recruit β-catenin to gene promoters [115,116] and both 1,25(OH)_2_D_3_ and VDR suppress β-catenin-stimulated LEF1/TCF-driven reporter activity [116,117]. The 1,25(OH)_2_D_3_ analogue EB1089 prevents the development of β-catenin-induced trichofolliculomas, while β-catenin activation in the absence of Vdr results in basal cell carcinomas [115]. Recently, Muralidhar and colleagues analyzed 703 primary melanoma transcriptomes and found that high tumor *VDR* expression is associated with upregulation of pathways mediating antitumor immunity and downregulation of proliferative pathways, notably Wnt/β-catenin [118]. Functional validation in vitro showed that 1,25(OH)_2_D_3_ inhibits the expression of Wnt/β-catenin pathway genes. These results suggest that 1,25(OH)_2_D_3_/VDR inhibits the pro-proliferative and immunosuppressive Wnt/β-catenin pathway in melanoma and that this is associated with less metastatic disease and stronger host immune responses [118].

Salehi-Tabar and colleagues have reported that *VDR* knockdown induces, while 1,25(OH)_2_D_3_ inhibits, β-catenin binding to and activation of *c*-*MYC* promoter in head and neck squamous cell carcinoma [119]. In this neoplasia, two vitamin D hydroxyderivatives, 20(OH)D_3_ and 1,20(OH)_2_D_3_, interfere with β-catenin nuclear translocation [120]. In a recent study, Rubin and colleagues analyzed the antitumor effects of 1,25(OH)_2_D_3_ and mitotane, the only chemotherapeutic agent available for adrenocortical carcinoma treatment. These authors reported a reduction in adrenocortical carcinoma cell growth and migration in response to either of the two agents, which is stronger when they are combined [121]. 1,25(OH)_2_D_3_ triggers a decrease in β-catenin RNA and nuclear protein levels, and both 1,25(OH)_2_D_3_ and mitotane induce RNA expression of the Wnt inhibitor *DKK1*, with a more marked effect with the combined treatment, although neither of them can reduce expression of the Wnt target gene *c*-*MYC* [121].

Vitamin D deficiency has been shown to promote hepatocellular carcinoma growth in *Smad3*^+/-^ mice via upregulation of toll-like receptor 7 expression and β-catenin activation and, accordingly, vitamin D supplementation reduced β-catenin levels [122]. In contrast, Matsuda and colleagues reported that neither dietary supplements of vitamin D nor treatment with vitamin D analogues affect tumor formation or growth in a mouse model of hepatocarcinogenesis induced by mutant β-catenin and c-*MET* overexpression. Hence, they questioned the utility of vitamin D for hepatocellular carcinoma therapy in that setting [123].

In summary, available data show that 1,25(OH)_2_D_3_ and its analogues interfere with Wnt/β-catenin signaling in a variety of human solid tumors using mechanisms that mostly resemble those observed in CRC cells (Table 1).

## 3. Antagonism of 1,25(OH)_2_D_3_/VDR Signaling by the Wnt/β-Catenin Pathway

The abovementioned data indicate that 1,25(OH)_2_D_3_ antagonizes Wnt/β-catenin signaling in several neoplasias. However, the interplay between both pathways is a two-way road, that is, activation of the Wnt/β-catenin pathway may also result in 1,25(OH)_2_D_3_/VDR inhibition.

VDR is the only high affinity receptor for 1,25(OH)_2_D_3_ and mediates most if not all 1,25(OH)_2_D_3_ effects. Thus, cellular VDR expression is the main determinant of 1,25(OH)_2_D_3_ action and its downregulation leads to 1,25(OH)_2_D_3_ unresponsiveness. VDR is expressed in most normal human cell types and tissues, but also in cancer cell lines and tumors of diverse origins. In line with the antitumor effects of 1,25(OH)_2_D_3_ observed in several neoplasias, high VDR expression in human cancer is usually a hallmark of good prognosis [29,31,36,37,124]. VDR expression and activity is regulated transcriptionally, posttranscriptionally by several microRNAs (miRs), and posttranslationally (via phosphorylation, ubiquitination, acetylation, and sumoylation) [125].

### 3.1. Repression of VDR by Snail Transcription Factors

Wnt/β-catenin signaling is known to promote EMT through upregulation of the expression and activity of key EMT transcription factors such as Snail1, Snail2, Zeb1 and Twist1 by several mechanisms [126,127]. Snail1 and Snail2 are phosphorylated by GSK-3β and tagged for β-TrCP-mediated ubiquitination and subsequent proteasomal degradation [128,129,130]. Thus, GSK-3β inhibition in response to Wnt/β-catenin signaling results in Snail1 and Snail2 protein stabilization. Inhibition of GSK-3β also increases *SNAI1* transcription via NFκB activation [131]. Furthermore, the Wnt/β-catenin target gene *AXIN2* contributes to Snail1 protein stabilization in breast cancer cells by regulating GSK-3β localization. When levels of AXIN2 increase in response to β-catenin/TCF signaling, GSK-3β is exported from the nuclear compartment leaving Snail1 in its non-phosphorylated transcriptionally active form [132]. In addition, induction of *SNAI2* RNA levels by Wnt3 has been described in breast cancer cells [133].

**Figure 2:**
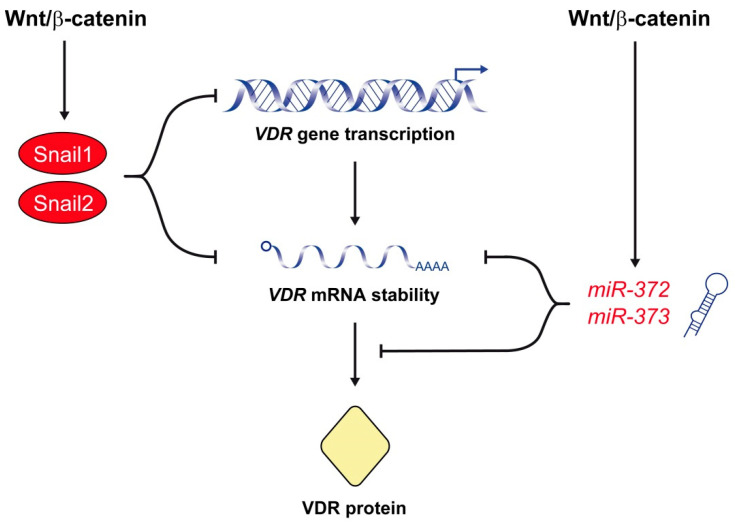
The Wnt/β-catenin signaling pathway represses VDR expression. A major mechanism of this effect is the upregulation of Snail1 and Snail2, which repress *VDR* gene transcription and decrease *VDR* RNA half-life. The Wnt/β-catenin pathway also antagonizes 1,25(OH)_2_D_3_/VDR signaling by the upregulation of *miR*-*372* and *miR*-*373*, which reduce the level of VDR RNA and protein.

Interestingly, Snail1 and Snail2 are the best-characterized transcriptional repressors of the human *VDR* gene. Our group demonstrated that Snail1 represses the expression of *VDR* by two mechanisms [83] (Figure 2). On the one hand, Snail1 inhibits *VDR* gene transcription by binding to three E-box sequences in its promoter. On the other, Snail1 reduces *VDR* RNA half-life. As a consequence, Snail1 strongly decreases the level of VDR RNA and protein and the cellular response to 1,25(OH)_2_D_3_ [83,84]. Moreover, forced expression of Snail1 in human CRC cells prevents the upregulation of E-cadherin and the subsequent cell differentiation triggered by 1,25(OH)_2_D_3_. Therefore, by repressing *VDR* and *CDH1*/E-cadherin genes, Snail1 abolishes 1,25(OH)_2_D_3_ action and favors the accumulation of β-catenin in the nucleus and the transcription of β-catenin/TCF target genes [83,84]. Later, Snail2 was found to also inhibit *VDR* gene expression in CRC cells through the same E-boxes in the promoter used by Snail1 (Figure 2). Actually, both transcription factors present an additive repressive effect on the *VDR* gene [134]. *SNAI1* and/or *SNAI2* upregulation is observed in 76% of human CRC and is associated with *VDR* downregulation [83,134,135,136]. Not surprisingly, the lowest *VDR* RNA levels are found in tumors with upregulation of both *SNAI1* and *SNAI2* genes [134]. *VDR* expression is also reduced in normal colonic tissue surrounding the tumor, which suggests that Snail1 expression in tumor cells promotes the secretion of factors that reduce *VDR* expression in neighboring normal cells [137]. In addition to CRC cells, Snail1 and Snail2 also repress *VDR* gene expression and antagonize the antitumor action of 1,25(OH)_2_D_3_ in human osteosarcoma and breast cancer cells [138,139]. Knackstedt and colleagues showed that downregulation of Vdr observed in the colon of a colitis mouse model is associated with an increase in the expression of Snail1 and Snail2 [140]. Altogether, these results support that activation of the Wnt/β-catenin pathway upregulates Snail1 and Snail2, which antagonizes 1,25(OH)_2_D_3_/VDR signaling by inhibiting *VDR* gene expression.

### 3.2. Posttranscriptional Repression of VDR by miRNAs

A novel, recently described mechanism of Wnt/β-catenin-mediated antagonism of 1,25(OH)_2_D_3_/VDR signaling involves the *miR*-*372/373* cluster (Figure 2). *miR*-*372/373* expression is induced by β-catenin/TCF in several human cancer cell lines through three TCF/LEF binding sites located in its promoter region [141]. Accordingly, this cluster of stem cell-specific miRs is dysregulated in various cancers, particularly in CRC due to the constitutive activation of the Wnt/β-catenin pathway [142,143,144]. Wang and colleagues have shown that overexpression of *miR*-*372/373* enhances the stemness of CRC cells and promotes their self-renewal, chemotherapy resistance and invasive potential [145]. These authors found that overexpression of *miR*-*372/373* results in upregulation of stemness-related pathways, e.g., Nanog and Hedgehog and, conversely, downregulation of differentiation-related pathways, e.g., NFκB, MAPK/ERK, and VDR. Interestingly, they demonstrated that *miR*-*372/373* overexpression leads to reduced expression of VDR RNA and protein in CRC cells, which contributes to the maintenance of the cancer stem cell phenotype [145]. These data suggest that the Wnt/β-catenin pathway also inhibits VDR expression through the induction of *miR*-*372/373*.

## 4. Conclusions

The Wnt/β-catenin pathway is frequently overactivated in cancer and promotes tumorigenesis, which makes it an attractive candidate for therapeutic intervention. The active vitamin D metabolite 1,25(OH)_2_D_3_, a major regulator of the human genome, cooperates with the Wnt/β-catenin pathway in the physiological control of tissues and organs such as bone, skin, and brain. Conversely, 1,25(OH)_2_D_3_ attenuates aberrant activation of the Wnt/β-catenin pathway that takes place in most CRC and in a variable proportion of other solid tumors. To do this, 1,25(OH)_2_D_3_ modulates a series of genes and mechanisms acting at different levels of the Wnt/β-catenin pathway that vary among cancer types. 1,25(OH)_2_D_3_ does not completely block the pathway but rather reduces its overactivation. This probably helps to maintain the physiological effects of Wnt/β-catenin in healthy organs, with few toxic side-effects. As expected from two crucial regulators of the organism and its necessary homeostasis, 1,25(OH)_2_D_3_ action is counterbalanced by Wnt factors.

The multilevel antagonistic action of 1,25(OH)_2_D_3_ on aberrantly activated Wnt/β-catenin signaling strongly supports the therapeutic utility of vitamin D compounds in cancer prevention and treatment.

## Figures and Tables

**Table 1:** Mechanisms of Wnt/β-catenin pathway interference by 1,25(OH)_2_D_3_ in solid cancers.

Cancer Type	Mechanism of Antagonism	Reference
Colorectal carcinoma	Increase of VDR/β-catenin interaction	[45]
	Upregulation of *CDH1*/E-cadherin	[34,45,50,51,52,83,85,87]
	Reduction of nuclear β-catenin	[45,77,84,87]
	Upregulation of *DKK1*	[54]
	Upregulation of *TCF7L2*/TCF4	[74]
	Upregulation of *AXIN1*	[76]
	Repression of *IL1B* (macrophages)	[78]
Breast carcinoma	Upregulation of *CDH1*/E-cadherin	[53]
	Reduction of active β-catenin	[102]
	Upregulation of *DKK1*	[102]
Ovarian carcinoma	Repression of *CCAT2* lncRNA	[106]
Kaposi’s sarcoma	Increase of VDR/β-catenin interaction	[109]
	Upregulation of VE-cadherin	[109]
	Upregulation of *DKK1*	[109]
Renal carcinoma	Increase of VDR/β-catenin interaction	[110]
	Upregulation of *CDH1*/E-cadherin	[110]
Pancreatic carcinoma	Increase of LRP6 lysosomal degradation	[111]
Head and neck squamous cell carcinoma	Reduction of nuclear β-catenin	[120]
Adrenocortical carcinoma	Reduction of nuclear β-catenin	[121]
	Upregulation of *DKK1*	[121]
Hepatocellular carcinoma	Reduction of β-catenin level	[122]
